# Dietary Capsaicin Protects Cardiometabolic Organs from Dysfunction

**DOI:** 10.3390/nu8050174

**Published:** 2016-04-25

**Authors:** Fang Sun, Shiqiang Xiong, Zhiming Zhu

**Affiliations:** The Center for Hypertension and Metabolic Diseases, Department of Hypertension and Endocrinology, Daping Hospital, Third Military Medical University, Chongqing Institute of Hypertension, Chongqing 400042, China; sun_fang2007@163.com (F.S.); xionglliu@163.com (S.X.)

**Keywords:** chili pepper, capsaicin, TRPV1, metabolic syndrome, obesity, hypertension, diabetes

## Abstract

Chili peppers have a long history of use for flavoring, coloring, and preserving food, as well as for medical purposes. The increased use of chili peppers in food is very popular worldwide. Capsaicin is the major pungent bioactivator in chili peppers. The beneficial effects of capsaicin on cardiovascular function and metabolic regulation have been validated in experimental and population studies. The receptor for capsaicin is called the transient receptor potential vanilloid subtype 1 (TRPV1). TRPV1 is ubiquitously distributed in the brain, sensory nerves, dorsal root ganglia, bladder, gut, and blood vessels. Activation of TRPV1 leads to increased intracellular calcium signaling and, subsequently, various physiological effects. TRPV1 is well known for its prominent roles in inflammation, oxidation stress, and pain sensation. Recently, TRPV1 was found to play critical roles in cardiovascular function and metabolic homeostasis. Experimental studies demonstrated that activation of TRPV1 by capsaicin could ameliorate obesity, diabetes, and hypertension. Additionally, TRPV1 activation preserved the function of cardiometabolic organs. Furthermore, population studies also confirmed the beneficial effects of capsaicin on human health. The habitual consumption of spicy foods was inversely associated with both total and certain causes of specific mortality after adjustment for other known or potential risk factors. The enjoyment of spicy flavors in food was associated with a lower prevalence of obesity, type 2 diabetes, and cardiovascular diseases. These results suggest that capsaicin and TRPV1 may be potential targets for the management of cardiometabolic vascular diseases and their related target organs dysfunction.

## 1. Introduction

A lot of protective natural compounds had been found for their neuroprotective properties in preventing diseases and inflammation [1,2,3,4]. Chili peppers have become a vital part of culinary cultures worldwide and have a long history of use for flavoring, coloring, and preserving food, as well as for medical purposes. Although some people are intolerant to pungency because of the sensation of heat and pain in the oral cavity, as well as varying degrees of gastrointestinal side effects, there remain many loyal consumers of this original South American plant. The increased use of chili peppers in food is a major trend around the world [5]. Capsaicin, the pungent ingredient in chili peppers, is an indispensable condiment, and it has shifted from an industrialized purified product to a daily nutrient. The beneficial effects of capsaicin have been validated in experimental and population studies.

The receptor for capsaicin is called the transient receptor potential vanilloid subtype 1 (TRPV1). TRPV1 belongs to the transient receptor potential (TRP) family, which is a heterogeneous group of non-selective cation channels. Based on their structural homology, mammalian TRP channels can be divided into six subfamilies, including the TRP canonical (TRPC; TRPC1–7), TRP vanilloid (TRPV; TRPV1–6), TRP melastatin (TRPM; TRPM1–8), TRP mucolipin (TRPML; TRPML1–3), TRP ankyrin (TRPA; TRPA1), and TRP polycystin (TRPP; TRPP2, TRPP3, TRPP5) subfamilies [6]. It is well documented that TRP channels are involved in visual, auditory, taste, and pain signal transduction pathways. Emerging evidence indicates that TRP channels also participate in the regulation of cell survival and growth, mineral absorption, body fluid balance, gut motility, and cardiovascular function [7]. TRPV1 is a highly investigated TRPV subfamily member. In addition to its classical role in the nervous system, TRPV1 plays important roles in the maintenance of physiological homeostasis. Capsaicin is passively absorbed with greater than 80% efficiency in the stomach and upper portion of the small intestine and is transported by albumin in the blood [8]; therefore, it may extensively activate local TRPV1 channels in different organs or tissues to initiate a series of physiological effects.

## 2. Physiological Function of TRPV1

**Figure 1:**
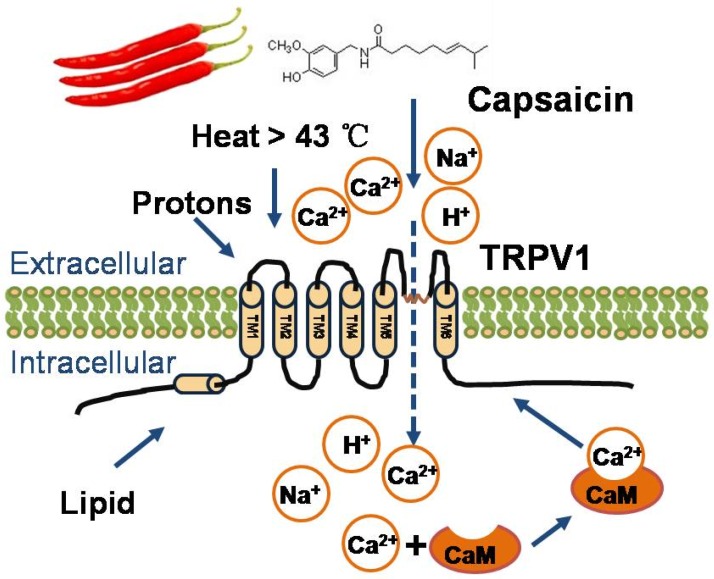
Structural and physiological function of TRPV1. TRPV1 is composed of six transmembrane domains. It has a short, pore-forming hydrophobic stretch between the fifth and sixth transmembrane domains. TRPV1 is activated by noxious heat (>43 °C), acid (pH < 5.9), voltage, and various lipids. Additionally, capsaicin activates TRPV1 and triggers cation influx and various subsequent physiological processes.

TRPV1 is widely expressed in the brain, sensory nerves, dorsal root ganglia, bladder, gut, and blood vessels [9,10]. TRPV1 is a ligand-gated non-selective cation channel, which is activated by multiple stimuli, including heat (>43 °C), voltage, low pH (<5.9), endogenous lipid molecules, and exogenous agonists, such as capsaicin [11] (Figure 1). Activation of TRPV1 leads to increased intracellular calcium levels and various physiological effects [12].

TRPV1 has prominent roles in inflammation, oxidative stress, and pain sensation [13]. Recently, emerging evidence suggests that TRPV1 also plays a critical role in the regulation of cardiovascular function and metabolic homeostasis. Activation of TPRV1 by its specific agonist capsaicin promotes endothelium-dependent vasodilation and subsequently contributes to lower blood pressure [14]. TRPV1 activation *in* *vivo* or in isolated perfused kidneys may increase the glomerular filtration rate and enhance renal sodium and water excretion [15,16]. TRPV1 was shown to be a potential target for the prevention of obesity because of its effect on energy balance [17,18]. Several studies found that activation of TRPV1 by capsaicin attenuated abnormal glucose homeostasis by increasing insulin secretion, insulin responses, and glucagon-like peptide 1 levels [19,20,21]. Furthermore, TRPV1 was shown as a regulator of growth factor signaling in the suppression of tumorigenesis [22], and its anti-cancer effect was also confirmed [23,24,25]. Furthermore, the TRPV1 receptor can be desensitized with high administration of capsaicin in nervous tissue [26], but whether this effect exists in cardiometabolic tissues was never tested. Furthermore, capsaicin also plays its effects in a receptor-independent manner. It reported that capsaicin could inhibit NF-kappa B and modulate adipocyte function in obese-mouse adipose tissues and isolated adipocytes which is independent on TRPV1 [27].

## 3. Roles of TRPV1 in Cardiometabolic Diseases

Mounting evidence indicates that TRPV1 activation by capsaicin is beneficial for the management of obesity, diabetes mellitus, cardiovascular diseases, various cancers, dermatological conditions, and neurogenic bladder [14,19,22,28,29].

### 3.1. Activation of TRPV1 by Capsaicin Prevents Obesity

Obesity is involved in the development of obesity-related disorders, such as diabetes, hyperlipidemia, fatty liver, and cardiovascular diseases. The results from both human and animal studies indicate that TRPV1 and its agonist capsaicin are involved in energy expenditure and may represent a potential strategy to treat obesity. Capsaicin inhibits obesity by regulating energy metabolism, reducing adipose tissue weight, and increasing lipid oxidation [17]. However, the underlying mechanisms are not fully understood.

Brown adipose tissue (BAT) is prominent in the regulation of energy expenditure and body fat [30,31]. TRPV1 activation by capsaicin can activate sympathetically-mediated BAT thermogenesis and reduces body fat [31]. Intragastric administration of capsiate, another TRPV1 agonist, also resulted in a time- and dose-dependent increase in integrated BAT sympathetic nerve activity and an increased the metabolic rate [32]. Capsinoids were found to increase the metabolic rate and enhance thermogenesis via gastrointestinal TRPV1 [33]. Endogenous TRPV1 ligands reduced food intake in wild-type mice but not in TRPV1-null mice [34]. The recruitment of catecholaminergic neurons by TRPV1 activation also contributed to the extra energy expenditure [35]. The results of a proteomic analysis revealed that approximately 23 protein spots, which are related to thermogenesis and lipid metabolism, were significantly altered in a capsaicin-fed rat liver. These proteins may represent potential targets of capsaicin to attenuate obesity [36]. Adipogenesis is the critical and original process of fatty adipose accumulation. Zhang *et al*. found that capsaicin treatment inhibited adipogenesis of 3T3-L1-preadipocytes *in vitro* and prevented high fat diet induced obesity [28]. Moreover, chronic activation of TRPV1 by dietary capsaicin reduced lipid deposition in the liver and alleviated abdominal obesity [37]. The mechanism of action was related to upregulated uncoupling protein 2 (UCP2) expression in hepatocytes [37]. Capsaicin treatment-enhanced lipolysis was associated with increased levels of hormone sensitive lipase, carnitine palmitoyl transferase-Iα and UCP2 [38]. Peroxisome proliferation activated receptor (PPAR) α, a key regulator of glucose and lipid metabolism, was also involved in the capsaicin treatment-induced decrease of levels of inflammatory cytokines and lipid droplet accumulation in the liver [39]. Dietary capsaicin supplementation in mice fed a high-fat diet confirmed that PPARγ expression in adipose tissue was decreased, whereas weight gain and visceral fat mass were blunted [12]. Similarly, Lee *et al*. showed that after topical application of capsaicin cream to the skin of mice fed a high-fat diet for 8 weeks, the mesenteric adipose tissue weighed less than that of the control obese mice [38]. The levels of plasma glucose, cholesterol, and triglycerides were also lower in the capsaicin-treated mice [38]. These beneficial effects of capsaicin treatment were associated with up-regulated adipokines, such as adiponectin and leptin [38].

The role of the TRPV1 channel deletion in obesity is controversial. Motter *et al*. [40] found that TRPV1-null mice exhibited a significantly greater thermogenic capacity. It suggested that inhibition of TRPV1-sensitive sensory seems to be protective. Lee *et al*. [41] demonstrated that TRPV1-null mice became more obese than wild-type mice while consuming a high-fat diet. The discrepancy between these findings could be associated with the different composition of high-fat diet (55% kcal% fat *vs*. 25.8% kcal% fat), feeding regimen, and the intervention time.

### 3.2. Activation of TRPV1 by Capsaicin Improves Glucose Homeostasis

Diabetes mellitus is one of the most important public health challenges. The prevention and treatment of diabetes mellitus, as well as a reduction in its microvascular and macrovascular complications, requires not only pharmacological approaches, but also a major integrated approach directed at societal and individual behavioral change. As a natural material and food ingredient, capsaicin has been extensively investigated because of its role in improving glucose homeostasis and alleviating diabetes.

The pathogenesis of type 2 diabetes is complex and involves oxidative stress, endoplasmic reticulum stress, and inflammation, facilitating insulin resistance and beta cell dysfunction. TRPV1 may promote insulin secretion via its calcium influx activity in β-cells; however, the mechanism by which TRPV1 affects insulin synthesis, degradation, and secretion is unknown [42]. Activation of TRPV1 alleviates insulin resistance and regulates glucose homeostasis by suppressing inflammation. Dietary capsaicin reduced obesity-induced insulin resistance and leptin resistance in mice [39,41]. This beneficial effect of capsaicin was due to the attenuation of inflammatory phenotypes and enhanced adiponectin expression in adipose tissue and liver, which are important peripheral tissues for insulin sensitivity [39]. Subsequently, dietary capsaicin significantly decreased fasting glucose/insulin and triglyceride levels, as well as the expression of inflammatory adipocytokine genes [43]. Moreover, insulin sensitivity during hyperglycemic states was enhanced in diabetic rats on a capsaicin diet [44]. Our previous studies showed that capsaicin supplementation ameliorates abnormal glucose homeostasis in diabetic mice via stimulating GLP-1 secretion [19]. Chronic capsaicin supplementation not only improved glucose tolerance and increased insulin levels but also lowered the daily blood profiles and increased plasma GLP-1 levels [19]. Both capsaicin and capsiate treatment reduced body weight gain, visceral fat accumulation, and serum leptin levels, and improved glucose tolerance without modulating energy intake in diabetic rats [44]. Both also protected β-cell mass by increasing proliferation and decreasing apoptosis [44]. As an exogenous agonist of TRPV1, capsaicin is a potential target for the management of type 2 diabetes.

### 3.3. Activation of TRPV1 by Capsaicin Alleviates Hypertension

As one of the leading risk factors for cardiovascular disease, the pathogenesis of hypertension refers to the imbalance between vasoconstriction and vasodilatation. Intracellular Ca^2+^ homeostasis is essential for vascular function and blood pressure regulation [45]. Disturbance of Ca^2+^ homeostasis contributes to vascular dysfunction and high blood pressure [46,47]. TRPV1 is involved in hypertension and its related target organ dysfunction.

Activation of TRPV1 by capsaicin exerts an anti-hypertension effect by promoting the release of calcitonin gene-related peptide (CGRP) from capsaicin-sensitive nerves and nitric oxide (NO) from endothelial cells. Acute administration of capsaicin induced a transient CGRP increase in the plasma and was accompanied by a decrease in blood pressure [48]. Yang *et al*. reported that activation of TRPV1 by dietary capsaicin up-regulated the phosphorylation of PKA and eNOS and, therefore, the bioavailability of NO in endothelial cells [14]. Long-term capsaicin treatment enhanced endothelium-dependent relaxation and lowered blood pressure in genetically hypertensive rats [14]. In addition, the release of CGRP contributed to the hypotensive effect of chronic capsaicin consumption, but to a lesser degree [14]. Recently, the inhibition of L-type Ca^2+^ channels in rat aortic smooth muscle cells was identified as the mechanism underlying capsaicin-induced relaxation [49]. TRPV1 activation prevented the salt-induced increase in blood pressure in Dahl salt-resistant rats [50]. However, the expression and function of TRPV1 were compromised in Dahl salt-sensitive rats, which rendered the Dahl salt-sensitive rats susceptible to salt load in terms of blood pressure regulation [50]. Chronic dietary capsaicin ameliorated excess salt consumption-induced vascular dysfunction and nocturnal hypertension by inhibiting vascular oxidative stress in a TRPV1-dependent manner [51]. Urinary sodium excretion was much higher in mice on a high salt diet plus capsaicin supplementation compared to mice fed only a high-salt diet [52]. This natriuretic effect of TRPV1 activation by capsaicin contributed to the lower blood pressure in mice fed a high salt diet. Furthermore, the general aversive behavior to salt caused by TRPV1 contributed to a lower dose of salt intake [53]. Marshall *et al*. [54] showed that TRPV1 deletion could protect against obesity-induced hypertension. Our studies demonstrated that the activation of TRPV1 by dietary capsaicin can attenuate genetic and high-salt diet induced hypertension [14,52]. Thus, the differences in experimental design and interventions could be responsible for this discrepancy.

### 3.4. Activation of TRPV1 Antagonizes Dysfunction of Cardiometabolic Organs

**Figure 2:**
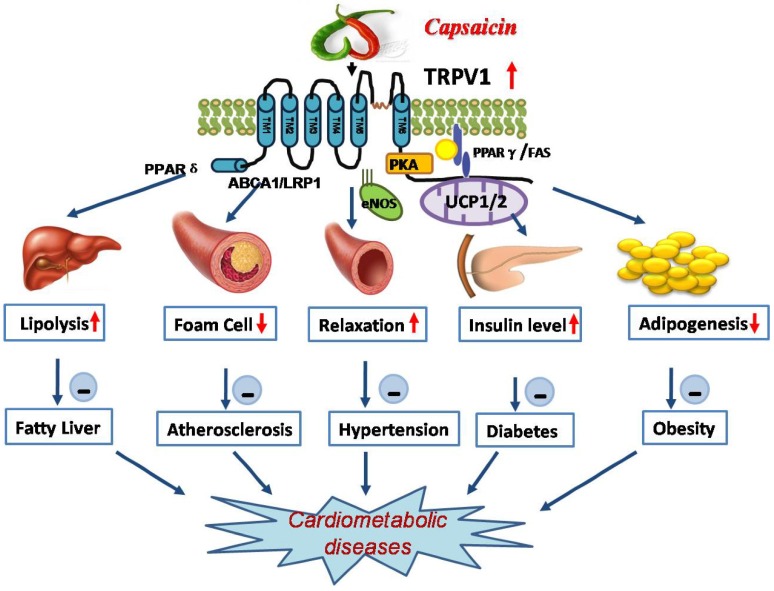
Favorable effects of capsaicin on cardiometabolic disease or related target organ damage. Activation of TRPV1 by dietary capsaicin plays a critical role in the regulation of lipid and glucose metabolism and vascular function, including the promotion of lipolysis by activating PPARδ, the improvement of vasodilation by increasing eNOS expression, the upregulation of insulin levels by activating PKA, the inhibition of foam cell formation by regulating ABCA1 and LRP1 levels, and the suppression of adipogenesis by activating PPARγ. Therefore, dietary capsaicin can alleviate fatty liver, atherosclerosis, hypertension, diabetes, and obesity. Dietary capsaicin has potential benefits for cardiometabolic diseases in the population.

Dietary capsaicin has favorable effects on obesity, diabetes, hypertension, and metabolic syndrome. Conceivably, activation of TRPV1 may alleviate cardiometabolic organs dysfunction, including ameliorating atherosclerosis, cardiac hypertrophy, non-alcoholic fatty liver, and stroke risk (Figure 2).

Capsaicin-rich diets have been found to improve lipid metabolism, and capsaicin supplementation reduced diet-induced hypertriglyceridemia in rodents [37,55]. Activation of TRPV1 by capsaicin-ameliorated non-alcoholic fatty liver disease in mouse models [37], and TRPV1-mediated induction of PPARδ and UCP2 likely played a role in this effect [37]. The lipoprotein lipase activity was higher in adipose tissues following capsaicin administration [56]. Dietary capsaicin also slows atherogenesis, an effect that may reflect a favorable impact of TRPV1 activation on foam cells. Ma *et al*. found that TRPV1 activation significantly inhibited foam cell formation by increasing ATP-binding cassette transporter A1 expression and reducing low-density lipoprotein-related protein 1 expression [57]. Chronic activation of TRPV1 by capsaicin supplementation reduced atherosclerotic lesions in the aorta from high-fat diet fed ApoE−/− mice but not from ApoE−/−TRPV1−/− mice [57]. The liver X receptor α also played a critical role in TRPV1-activation-conferred protection against oxLDL-induced lipid accumulation and TNF-α-induced inflammation in macrophages [58]. Recently, we showed that TRPV1 activation antagonized coronary lesions by alleviating endothelial mitochondrial dysfunction and enhancing the activity of the PKA/UCP2 pathway [59]. Ultimately, this beneficial effect of TRPV1 activation prolonged the mean survival time of atherosclerotic mice [59].

In the heart, TRPV1 activation blunted cardiac hypertrophy and fibrosis [60,61]. High-salt intake-induced cardiac hypertrophy and fibrosis were characterized by a significant enhancement of heart weight, decreased heart function, and increased collagen deposition [60]. These alterations were related to the downregulation of PPARδ and UCP2 expression, the upregulation of iNOS production, and increased oxidative/nitrotyrosine stress [60]. Oxidative phosphorylation and the enzyme activity of the mitochondrial complex I were impaired in TRPV1 knockout or high-salt diet fed mice [61]. Indeed, these adverse effects of long-term high-salt intake were blunted by chronic capsaicin supplementation in a TRPV1-dependent manner [60,61]. Capsaicin-rich diets also attenuated pressure overload- and angiotensin II-induced cardiac hypertrophy and fibrosis [62].

Dietary capsaicin was shown to significantly delay the onset of stroke and increase the survival time of spontaneously hypertensive stroke-prone rats [63]. This anti-stroke effect of capsaicin was related to the enhanced relaxation of cerebral arteries and reversed hypertrophy of cerebral arterioles [63]. The neuroprotective effects of endocannabinoids were partially mediated by TRPV1, which afforded protection to the blood-brain barrier during ischemic stroke [64].

Diabetic vascular complication is a major cause of death and disability. Diabetes mellitus induces vascular endothelial dysfunction via several mechanisms, including excessive ROS generation following mitochondrial disturbance and the impairment of eNOS activity, finally leading to oxidative stress and endothelium dysfunction. UCP2 is a physiological regulator of mitochondrial ROS generation and may contribute to the prevention of diabetes and its related complications. Sun *et al*. found that upregulation of UCP2 by capsaicin decreased ROS production and increased NO bioavailability [65]. Chronic dietary capsaicin suppressed vascular oxidative stress and improved endothelium-dependent relaxation in diabetic mice [65]. By contrast, the expression of TRPV1 was decreased in diabetic mesenteric arteries, which was associated with impaired capsaicin-induced vasodilatation [66].

The gastrointestinal tract releases various gut hormones, such as cholecystokinin, ghrelin, peptide YY, and GLP-1, which participate in the stimulation of gastrointestinal function, the maintenance of energy homeostasis and metabolism [67]. Recently, several studies reported that gut hormones are involved in the pathogenesis of diabetes, obesity and hypertension [68,69,70]. Dietary capsaicin stimulated the intestinal mucosal afferent nerves and increased intestinal blood flow, which may affect the physiological function of the gastrointestine [71]. GLP-1 plays a principal role in the regulation of glucose metabolism by modulating insulin secretion and activating the gut-brain-periphery axis [72]. We found that TRPV1 receptors are present in GLP-1-expressing intestinal cells and that activation of TRPV1 stimulated GLP-1 release via a Ca^2+^-dependent mechanism [19]. Chronic dietary capsaicin lowered blood glucose levels and improved glucose homeostasis in db/db mice [19]. Human studies also demonstrated that a single meal with capsaicin increased plasma GLP-1 concentrations and tended to decrease the plasma ghrelin concentrations during the postprandial phase [73]. Ghrelin is a stimulator of food intake and a centrally-acting orexigenic hormone. Activation of the capsaicin-sensitive vago-vagal reflex pathway was involved in ghrelin stimulated gastric motility [74]. Nesfatin-1, a newly identified hormone, belongs to a family of anorexigenic peptides. Nesfatin-1-induced protection was attenuated by pretreatment with the TRPV1 receptor inhibitor capsazepine [75].

## 4. Beneficial Effects of Dietary Capsaicin Consumption in Humans

The favorable effects of spices and their bioactive ingredients, such as capsaicin, have been documented in many experimental studies. Population studies also confirm the beneficial effects of capsaicin on human health (Table 1).

In a large prospective cohort study, the habitual consumption of spicy foods was inversely associated with both total and certain cause-specific mortality among both men and women after adjustment for other known or potential risk factors [76]. Inverse associations were also observed for deaths due to cancer, respiratory diseases, and ischemic heart diseases [76]. Compared with people who ate spicy foods less than once a week, the people who ate spicy foods almost every day had a 14% lower risk of death [76].

Epidemiologic data also showed that the consumption of foods containing capsaicin is associated with a lower prevalence of obesity, type 2 diabetes, and cardiovascular diseases [77,78]. Obesity is prominent risk factor for diabetes and cardiovascular diseases. Increased energy expenditure, enhanced lipid oxidation, and reduced appetite are potentially beneficial for weight management. Dietary red pepper was shown to suppress energy intake and modify macronutrient intake through its effects on appetite and energy expenditure. The ingestion of red pepper decreased appetite and subsequent protein and fat intake [79]. Red pepper also increased diet-induced thermogenesis and lipid oxidation [80]. The consumption of capsaicinoids increased energy expenditure by approximately 50 kcal/day [13], and this would produce clinically significant levels of weight loss [81]. However, there were inconsistent results for these outcomes. Some studies did not show any effect on substrate oxidation, energy expenditure or appetite following one meal or long-term administration in the form of capsules, juice, or supplements [82]. These differences may be a result of race, district, and dietary habits. Additionally, the range of dosage, method of administration and composition are widely varied. Therefore, a dietary capsaicin test is in urgent need of a unified standard.

The potential role of capsaicin in glucose homeostasis has been validated in animal experiments, although evidence for capsaicin regulating glucose metabolism in humans is relatively limited. In a large prospective study, the inverse association of daily spicy food consumption with death due to diabetes was observed in people who habitually ate fresh chili peppers [76]. In healthy human subjects, capsaicin treatment increased glucose absorption from the gastrointestinal tract and increased glucagon release during glucose loading tests [83]. The increased release of glucagon was proposed to be independent of insulin release after glucose loading. A low dose of capsaicin stimulated glucose absorption from the gastrointestinal tract in healthy human subjects and promoted the mobilization of glycogen via the stimulation of capsaicin-sensitive afferent nerves [83]. This result indicates that capsaicin-sensitive afferent nerves exert an important role in glucose utilization. Furthermore, topical capsaicin treatment significantly relieved pain at 12 weeks in patients with diabetic peripheral neuropathy [84].

Whether capsaicin is favorable for human cardiovascular health needs further study. The habitual consumption of spicy foods was inversely associated with deaths due to ischemic heart diseases [76]. Capsaicin may inhibit ADP-induced platelet aggregation [85,86]. The transdermal administration of capsaicin improved the ischemic threshold and exercise time in patients with stable coronary disease [87]. NO levels were also increased in the blood, and NO-mediated vasodilation may contribute to this clinical benefit. In combination with isoflavone, capsaicin significantly reduced both systolic and diastolic BP in hypertensive patients with alopecia [88]. This was likely associated with elevated serum levels of insulin-like growth factor-I, which has an antihypertensive effect [88].

Capsiate, a recently identified non-pungent capsaicin analog, presents a promising alternative for those who abstain from capsaicin-containing foods due to pungency. It provides a more acceptable compound for clinical trials that study the potential mechanism of dietary capsaicin on cardiometabolic organ protection in the population [89].

## 5. Conclusions

Capsaicin is not only a dietary nutrient but also a natural bioactive food ingredient. Capsaicin is involved in thermogenesis, lipid metabolism, the inflammatory response, and oxidative stress. These effects of capsaicin reduce adipogenesis, alleviate insulin resistance, ameliorate vascular dysfunction and regulate glucose homeostasis. These pathophysiologic processes are responsible for the pathogenesis of cardiometabolic diseases, such as obesity, hypertension, dyslipidemia, diabetes and atherosclerosis. Capsaicin plays a potential role in cardiometabolic protection through the activation of TRPV1 in different target organs or tissues, which suggests that TRPV1 may be a promising target for the management of cardiometabolic diseases. It is necessary to identify a more acceptable way to clarify the association between the dosage of dietary capsaicin and the effect on cardiometabolic protection to finally reach a consensus on the daily usage of capsaicin or its derivatives.

## Figures and Tables

**Table 1:** The effects of capsaicin on cardiometabolic diseases in human studies.

Involved Diseases	References	Effect of Capsaicin	Underlying Mechanism
Obesity	[79,80,81]	+	increase energy expenditure, lipid oxidation and sympathetic nervous system activity; decrease appetite and subsequent protein and fat intake
	[82]	N	-
Type 2 diabetes	[83]	+	increase glucose absorption and glucagon release
Diabetic peripheral neuropathy	[84]	+	stimulation of capsaicin-sensitive afferent nerves
Cardiovascular diseases			
Coronary disease	[86,87] [88]	+	inhibit ADP-induced platelet aggregation; increase NO levels in the blood and NO mediated vasodilation
Hypertension			
		+	elevate serum levels of insulin-like growth factor-I

Note: +, beneficial effect; N, no effect. [79,80,81,86,87]: randomized controlled trial, [82]: meta-analysis, [83]: comparative study, [84]: randomized, double-blind and parallel-group trial, [88]: case-control studies.
